# Case Report: Acute large bowel obstruction with actinomycosis of the sigmoid colon mimicking neoplasm

**DOI:** 10.12688/f1000research.151907.1

**Published:** 2024-06-21

**Authors:** Mohamed Hajri, Rami Zouari, Ines Mallek, Dhouha Bacha, Rached Bayar, Sana ben slama

**Affiliations:** 1 Faculty of Medicine, Universite de Tunis El Manar, Tunis, Tunis, Tunisia; 2 Visceral surgery, University Hospital Center Mongi Slim, La Marsa, Tunis, Tunisia; 3 Pathology, University Hospital Center Mongi Slim, La Marsa, Tunis, Tunisia

**Keywords:** Abdominal actinomycosis, actinomyces, acute intestinal obstruction, antibiotic therapy, surgery.

## Abstract

**Introduction:**

Actinomycosis is an uncommon inflammatory bacterial disease caused by Actinomyces species, especially Actinomyces Israeli. Abdominopelvic forms are relatively rare and may involve the colon as a solid mass, mimicking a malignant tumor.

**Case presentation:**

A 68-year-old Tunisian man, with a history of diabetes, hypertension, penicillin allergy, and renal failure, presented to the emergency department with abdominal pain, vomiting, and bowel obstruction. CT scan showed an acute intestinal obstruction upstream with obstructive tissular mass at the sigmoid colon. Emergency surgery revealed a sigmoid mass and a pre-perforative cecum. Subtotal colectomy was performed, with ileostomy and distal end closure. Histological examination confirmed Actinomyces infection. The patient was then placed on long-term doxycycline and Bactrim, with no recurrence over a 9-month follow-up period.

**Conclusion:**

Abdominal actinomycosis, though rare, presents diagnostic challenges. It can be mistaken for malignancy, leading to unnecessary surgery in non-complicated cases, since it is effectively treated by antibiotics. In complicated cases, a combined approach involving both surgery and antibiotic therapy is necessary until the infection is completely eradicated.

## Introduction

Actinomycosis is an uncommon inflammatory bacterial disease caused by Actinomyces Israeli, a Gram-positive anaerobic bacterium typically found in the digestive and genital tracts. This condition is often mistaken for a tumor or presents as an inflammatory mass. It can also lead to the formation of abscesses. ^1^ The progression is slow and insidious, with local inflammation extending across different organs without confinement to a single one. ^2^ Actinomyces typically colonizes the oral cavity, bronchi, gastrointestinal and female genital tracts. In the gut, it preferentially involves the stagnated zones, notably the caecum, the appendix, and the sigmoid colon. Clinical manifestations and radiological findings are nonspecific. ^3^ Since acute and complicated forms require early treatment, most forms are diagnosed postoperatively. In this case report, we present a rare occurrence of colonic actinomycosis mimicking neoplasm and causing acute large bowel obstruction.

## Case presentation

**Figure 1.**
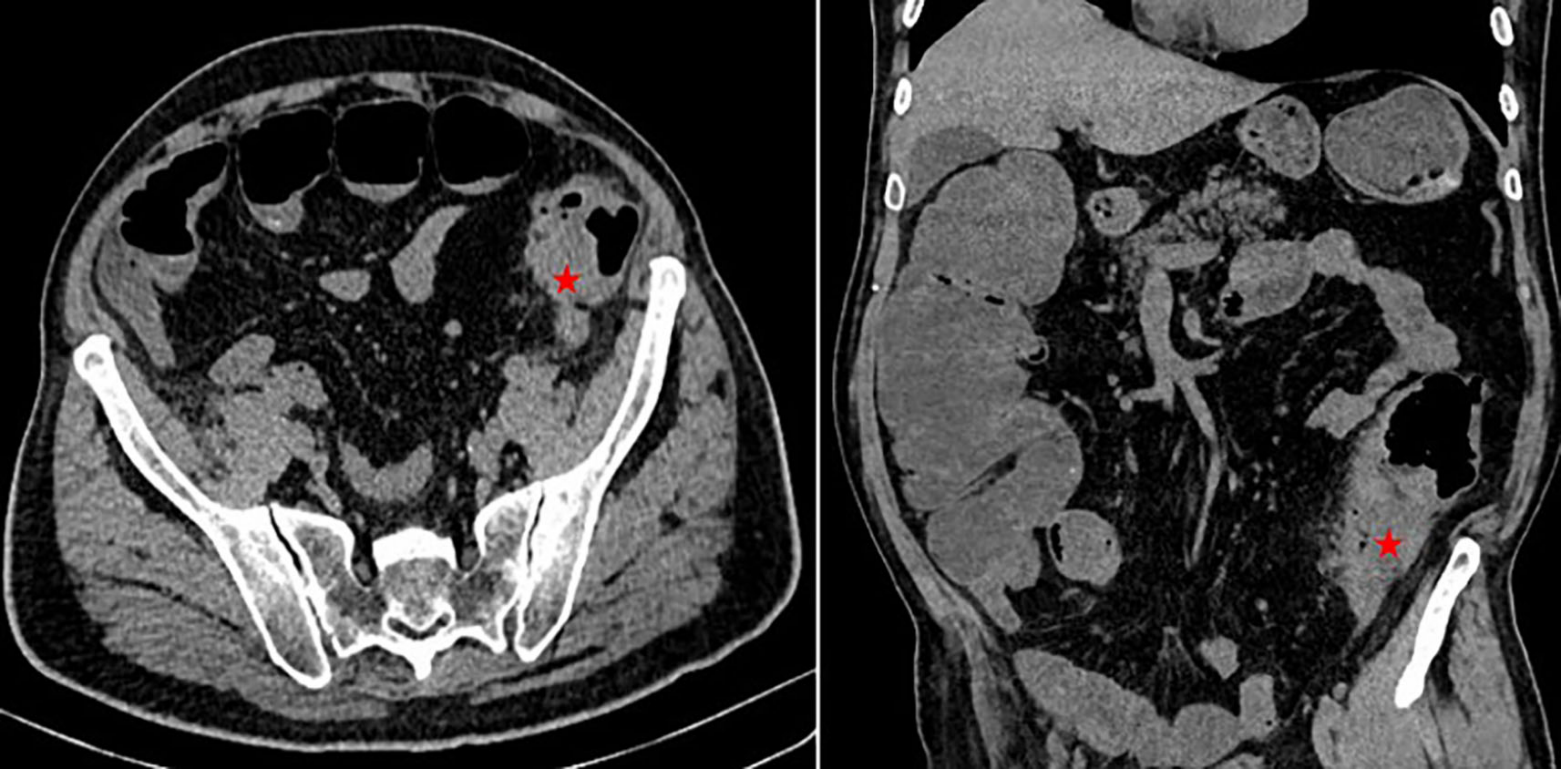
Abdominal CT scan showing acute intestinal obstruction with an obstructive tissular mass at the sigmoid colon (red asterixis).

**Figure 2.**
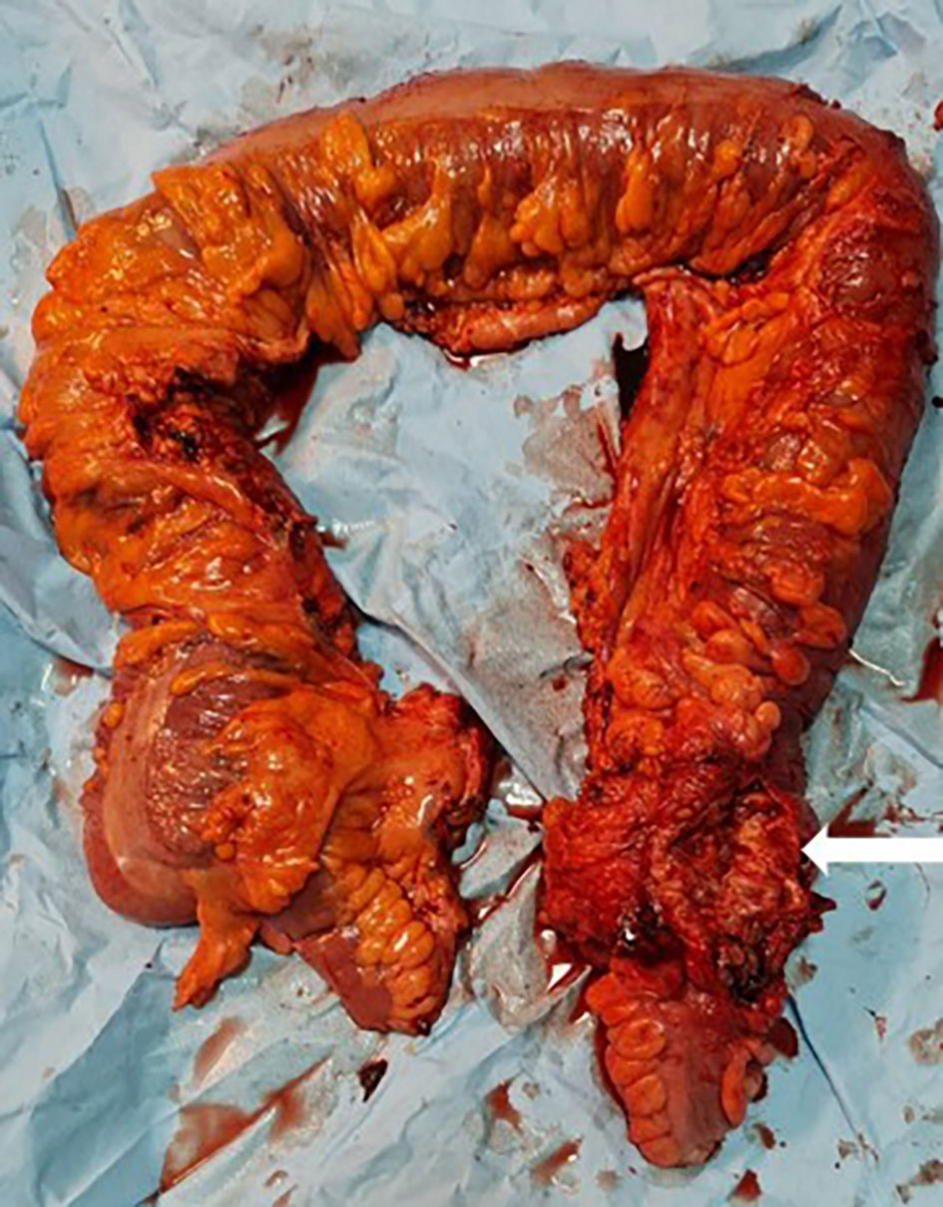
Total colectomy specimen with a 7 cm obstructive solid mass of the sigmoid colon (white arrow).

**Figure 3.**
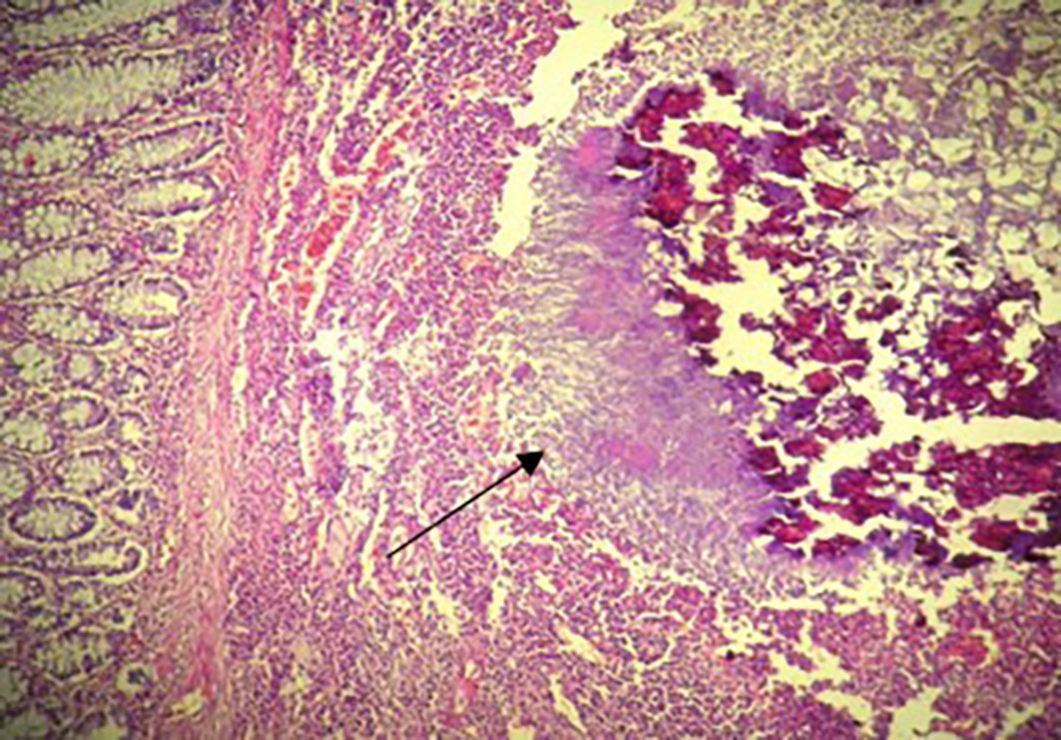
Histological examination: Colonic actinomycosis with spherical cluster of actinomyces and a suppurative inflammation at the periphery (H&E ×200).

**Figure 4.**
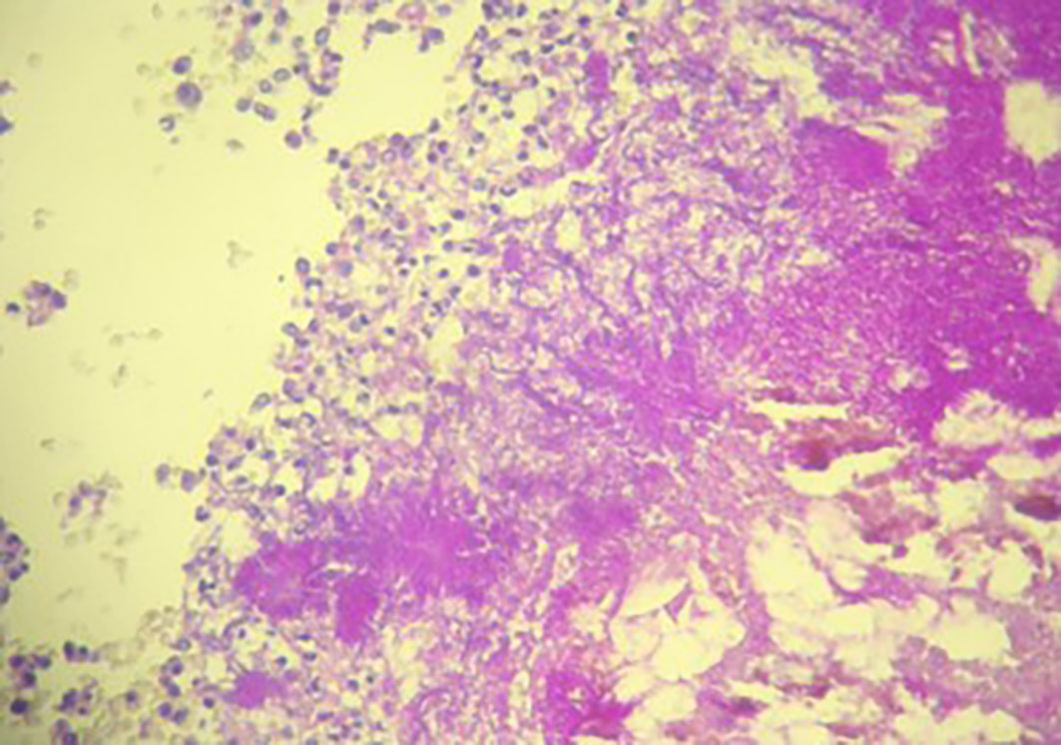
Densely packed filamentous bacteria arranged in clusters surrounded by polymorphic inflammatory cells (PAS Stain ×200).

A 68-year-old Tunisian man, with a history of diabetes, hypertension, penicillin allergy, and renal failure, with no prior surgical history, presented to the emergency department with abdominal pain, vomiting, and bowel obstruction. The patient reported a similar symptomatology over the last two months, which resolved spontaneously. He also complained of chronic abdominal discomfort. On examination, he was hemodynamically stable. There was no fever. The abdominal examination revealed a distended and resonant abdomen, tender throughout, with a palpable mass in the left iliac fossa. The laboratory tests were normal, except for a previously known renal failure. We followed up with an abdominal CT scan without contrast, which revealed an acute intestinal obstruction upstream of a suspected obstructive tissue process at the sigmoid colon, with associated satellite lymph nodes and a dilated cecum measuring 12 cm ( Figure 1). Emergency surgery was decided after a brief resuscitation. Exploration of the abdomen by midline laparotomy revealed a mass in the sigmoid loop, measuring 7 cm along its major axis, adherent to the omentum, the parietal peritoneum, and the posterior wall, with dilation of the entire upstream colonic frame. The cecum was dilated to 13 cm with a weakened, pre-perforative wall. We underwent a total colectomy with ileostomy and distal end closure ( Figure 2). The postoperative recovery was uneventful. Gross pathologic examination of the surgical specimen revealed a stenosing lesion of the sigmoid colon with ulcerated surface mucosa. Histological examination showed acute inflammatory reaction and abscess formations surrounding clumps of short branching basophilic filaments stained with PAS (Periodic Acid Schiff). Dense fibrosis was associated. There was no granulomatous inflammation ( Figures 3, 4). Actinomyces infection was confirmed. The patient was then placed on long-term doxycycline and Bactrim. Upon follow-up, he was seen regularly for 9 months. No recurrence has been diagnosed. The restoration of bowel continuity was postponed until completing a full year of antibiotic therapy.

## Discussion

Actinomycosis is a rare granulomatous inflammation caused by Actinomyces species, especially Actinomyces Israeli, gram-positive anaerobic bacteria that are part of the normal human flora, colonizing the oral, digestive, and urogenital tracts. ^4^^,^^5^

Actinomyces species have low virulence potential and require mucosal barrier disruption. This can occur after surgery, trauma, or in the presence of a foreign body, or in immunosuppression situations. ^6^

All tissues may be infected, and we can distinguish four types of pre-ponderant infections, cervicofacial 50 to 60%, thoracic 15%, abdominopelvic 20%, and rarely disseminated disease. ^7^

Actinomycosis commonly occurs between the ages of 20 and 60 years old and affects men three times more than women. ^8^ Nevertheless, its incidence in women is increasing, associated with the rising use of intrauterine devices, reaching 75% of patients with pelvic actinomycosis in some studies. ^8^

Abdominopelvic forms can mimic malignant tumors due to their chronic evolution. They have no specific clinical presentation and patients can consult for various symptoms such as chronic abdominal pain, abdominal mass, nausea, vomiting, anorexia, weight loss, and bleeding. ^9^

This explains that it can evolve insidiously and manifest as a voluminous mass at the time of diagnosis.

Our case described an unusual presentation of abdominopelvic actinomycosis characterized by large bowel obstruction occurring in a 75-year-old patient with none of the risk factors cited above. It was due to a pseudotumoral sigmoid mass with a pre-perforative cecum.

Differential diagnoses in patients presenting with abdominal forms include appendicitis, diverticulitis, inflammatory bowel disease, tuberculosis, and bowel malignancies. ^10^

Being a chronic suppuration, abdominal actinomycosis leads to the formation of multiple adjacent abscesses and to a large inflammatory reaction that can potentially invade neighboring tissues, appearing as a locally advanced tumor. ^6^^,^^10^

In addition, being able to spread through hematogenous ways, actinomycosis may cause distant infections, mimicking distant metastasis. ^10^

The management of abdominopelvic actinomycosis depends on its presentation.

The diagnosis can be suspected on CT scan findings, and confirmed after undergoing CT-guided puncture where Actinomyces species can be identified.

In such cases, the patient will undergo long-term antibiotherapy, such as parenteral penicillin G, followed by oral penicillin V or amoxicillin for up to 12 months. Alternative antibiotics like Tetracycline, Erythromycin or Clindamycin can be given in patients with penicillin allergy.

Generally, the prognosis is favorable and treatment efficacy is verified through ultrasonography or computer tomography. ^8^^,^^11^^,^^12^

However, in most cases, actinomycosis is only diagnosed postoperatively. Indeed, confusion with a malignant mass, or manifestation in complicated forms, as in our observation, often leads to primary surgery.

According to the literature, actinomycosis involving the colon and presenting as acute abdomen or acute large bowel obstruction is rarely reported.

A review of the literature was conducted using the PubMed Database. We used “actinomycosis”, “colon”, “intestinal obstruction”, and “acute abdomen” as keywords. We excluded articles that reported extrinsic invasion of the colon, non-complicated colonic actinomycosis treated with antibiotics, and manuscripts not written in English.

A total of 15 articles were found between 1980 and 2024.

The most commonly affected colonic segments were the ascending colon and the transverse colon. In almost all reported cases, emergency surgery was performed and the diagnosis was made postoperatively. All patients received prolonged antibiotic therapy after surgery, with no reported recurrence.

In only one case, as detailed by Lin et al., ^13^ the diagnosis was made through endoscopic biopsy conducted during an episode of acute infectious colitis, suspected to be caused by actinomycosis infection. The patient was successfully treated with antibiotics and did not require surgery.

The details are summarized in Table 1.

**Table 1.** Literature review (1980-2024) of complicated colonic actinomycosis cases.

Year	Author	Age	M/F	Size	Localization	Presentation/Complication	Diagnosis	Treatment
2024	Our case	68	M	7 cm	Sigmoid colon	Acute large bowel obstruction	Postoperative anatomopathological examination	Surgery + antibiotic therapy
2023	Lyew et al. ^14^	48	F	7.7 × 4.8 × 4.5 cm	Transverse colon, small bowel, abdominal wall	Abdominal pain, epigastric mass	Postoperative anatomopathological examination	Surgery + antibiotic therapy
2021	Pamathy et al. ^6^	40	F	9.7 × 4.5 cm	Transverse colon + descending colon	Acute large bowel obstruction	Postoperative anatomopathological examination	Surgery + antibiotic therapy
2020	Morais-Kansaon et al. ^15^	46	F	3.2 × 3.6 × 2.8 cm	Transverse colon	Acute abdominal pain	Postoperative anatomopathological examination	Surgery + antibiotic therapy
2020	Jabi et al. ^16^	48	M	_	Sigmoid colon	Acute abdominal pain, fever, general health state deterioration	Postoperative anatomopathological examination	Surgery + antibiotic therapy
2019	Hui et al. ^17^	35	F	8 cm	Caecum	Abdominal discomfort, fever, right iliac fossa mass	Postoperative anatomopathological examination	Surgery + antibiotic therapy
2018	Yang et al. ^10^	55	F	_	Sigmoid colon	Colon perforation	Postoperative anatomopathological examination	Surgery + antibiotic therapy
2016	García-Zúñiga et al. ^18^	41	M	_	Distal ileum + ascending colon	Acute abdominal pain, fever, diarrhea, weight loss	Postoperative anatomopathological examination	Surgery + antibiotic therapy
2008	Valko et al. ^19^	38	F	10 cm	Sigmoid colon	Acute large bowel obstruction	Postoperative anatomopathological examination	Surgery + antibiotic therapy
2007	Saha et al. ^20^	_	_	_	Transverse colon	Acute abdominal pain	Postoperative anatomopathological examination	Surgery + antibiotic therapy
2006	Jung ^21^	27	F	6 × 7 cm	Sigmoid colon + small bowel + mesentery	Colon perforation	Postoperative anatomopathological examination	Surgery + antibiotic therapy
2005	Filippou et al. ^22^	72	F	5 × 5 cm	Caecum	Pericolic abscess	Postoperative anatomopathological examination	Surgery + antibiotic therapy
2005	Işık et al. ^23^	28	M	8 × 6 cm	Ascending colon	Acute abdominal pain + vomiting	Postoperative anatomopathological examination	Surgery + antibiotic therapy
2004	Bittencourt et al. ^24^	58	M	_	Caecum + distal ileum	Acute large bowel obstruction	Postoperative anatomopathological examination	Surgery + antibiotic therapy
2003	Lin et al. ^13^	45	F	_	Caecum (mass) Entire colon (colitis)	Acute abdominal pain, weight loss, diffuse colitis	Endoscopic biopsy	Antibiotic therapy
2000	T.C.A Ferrari et al. ^25^	56	F	3 cm 15 cm	Transverse colon Descending colon	Colon fistulization	Postoperative anatomopathological examination	Surgery + antibiotic therapy

In our particular case, emergency surgery was inevitable given the obstructive character of the sigmoid colon lesion. We underwent a total colectomy due to the pre-perforative lesions appearing on the cecum. Due to anatomopathological findings, he was prescribed long-term antibiotherapy based on doxycycline and Bactrim regarding his penicillin allergy.

Despite appropriate treatment, abdominal actinomycosis may recur and patients should be followed up. Currently, there is no standardized protocol for this monitoring. However, patients should at least undergo ultrasonography or computed tomography after treatment. ^12^

## Conclusion

Abdominal actinomycosis, though rare, presents diagnostic challenges. It can be mistaken for malignancy, leading to unnecessary surgery in non-complicated cases. The diagnosis should be considered when there is an abdominal mass with local invasion signs, whether or not an infectious syndrome is present. As a result, all efforts should be made to confirm the diagnosis. Once the diagnosis is certain through microbiological or pathological examinations, antibiotic treatment with penicillin should be started, lasting for six to 12 months, depending on the extent of the infection. This extended treatment duration helps reduce the risk of recurrence and often completely resolves the lesions. In complicated cases, a combined approach involving surgery and antibiotic therapy is necessary until the infection is completely eradicated.

### Ethical approval statement

Written informed consent was obtained from the patient for the publication of this case report and the accompanying images.

## Data Availability

No data are associated with this article.
