# Supplementary material for: Case Report: Acute large bowel obstruction with actinomycosis of the sigmoid colon mimicking neoplasm
Source: F1000Res. 2024 Aug 29;13:669. Originally published 2024 Jun 21. [Version 2] doi: 10.12688/f1000research.151907.2 (PMC11393526; doi:10.12688/f1000research.151907.2)
Supplement: Peer review history

# Reviewer report: Reviewer response for version 2

**DOI:** 10.5256/f1000research.170479.r318558

**Published:** 2024-09-12

**Authors:** Mhasisielie Zumu, Dr Arun RS

**Affiliations:** 1 Medical Gastroenterology, The Madras Medical Mission, Chennai, Tamil Nadu, India

The concerns have been addressed.

Are enough details provided of any physical examination and diagnostic tests, treatment given and outcomes?

Partly

Is the case presented with sufficient detail to be useful for other practitioners?

Partly

Is sufficient discussion included of the importance of the findings and their relevance to future understanding of disease processes, diagnosis or treatment?

Partly

Is the background of the case’s history and progression described in sufficient detail?

Partly

Reviewer Expertise:

Gastric actinomycosis

We confirm that we have read this submission and believe that we have an appropriate level of expertise to confirm that it is of an acceptable scientific standard.

# Reviewer report: Reviewer response for version 1

**DOI:** 10.5256/f1000research.166599.r311560

**Published:** 2024-08-21

**Authors:** Prajwal Dahal

**Affiliations:** 1 Grande International Hospital, Tokha, Kathmandu, India

I would like to congratulate the author for writing a comprehensive case report in a entity that is less encountered in clinical practice. The manuscript adds on our current knowledge of actinomycosis infection of bowel and  stimulates clinicians and radiologists to consider it as a differential of bowel mass. I suggest minor revision for betterment.

 1) Please provide values of vitals, blood parameters and serum urea/ creatnine at presentation

 2) In a case report, it is advised to limit number of reference to below 10. Kindly consider that.

Are enough details provided of any physical examination and diagnostic tests, treatment given and outcomes?

Yes

Is the case presented with sufficient detail to be useful for other practitioners?

Yes

Is sufficient discussion included of the importance of the findings and their relevance to future understanding of disease processes, diagnosis or treatment?

Yes

Is the background of the case’s history and progression described in sufficient detail?

Yes

Reviewer Expertise:

Radiology

I confirm that I have read this submission and believe that I have an appropriate level of expertise to confirm that it is of an acceptable scientific standard.

# Response

**Published:** 2024-08-29

**Authors:** Mohamed Hajri

**Affiliations:** General Surgery, University Hospital Center Mongi Slim, La Marsa, Tunis, Tunisia

Thank you very much for your positive feedback and constructive suggestions. I appreciate your time and effort in reviewing my manuscript.

1. I will include the values of vitals, blood parameters, and serum urea/creatinine at the time of presentation in the revised version.
2. I understand the recommendation to limit the number of references to below 10. However, many of the references have been used in the summary table to cite the different cases from the literature. Would it be feasible to keep them included? If you still recommend reducing the number of references, I would be willing to do so.

Thank you again for your valuable input.

# Reviewer report: Reviewer response for version 1

**DOI:** 10.5256/f1000research.166599.r299051

**Published:** 2024-07-22

**Authors:** Mhasisielie Zumu, Dr Arun RS

**Affiliations:** 1 Medical Gastroenterology, The Madras Medical Mission, Chennai, Tamil Nadu, India

1) Details on blood sugar controlled, alcohol used, smoking, oral hygiene and history of prolonged used of PPI may be mentioned, if present.  

 2) Serum HbA1c level at admission may be mentioned 

 3) No risk factor of immunosuppression?  - diabetes mellitus is present.

 4) Is the Actinomyces infection only in sigmoid colon or in caecum as well causing weakened, pre-perforative wall ?- what was the histopathology report ?

 5) "We underwent a total colectomy with ileostomy and distal end closure" - grammar check  

 6) The surgery done has been stated as subtotal colectomy in the abstract. However, subsequently it has been mentioned as total colectomy. please correct it

 7) Was sulphur granules looked for /found in histopathology?

 8) pre-preforative wall- terminology to be check

Are enough details provided of any physical examination and diagnostic tests, treatment given and outcomes?

Partly

Is the case presented with sufficient detail to be useful for other practitioners?

Partly

Is sufficient discussion included of the importance of the findings and their relevance to future understanding of disease processes, diagnosis or treatment?

Partly

Is the background of the case’s history and progression described in sufficient detail?

Partly

Reviewer Expertise:

Gastric actinomycosis

We confirm that we have read this submission and believe that we have an appropriate level of expertise to confirm that it is of an acceptable scientific standard, however we have significant reservations, as outlined above.

# Response

**Published:** 2024-08-15

**Authors:** Mohamed Hajri

**Affiliations:** General Surgery, University Hospital Center Mongi Slim, La Marsa, Tunis, Tunisia

I am grateful for the time and effort you and the reviewers have invested in offering valuable feedback on my manuscript. I sincerely appreciate the reviewers' insightful comments on our paper. I have made revisions to incorporate most of the suggestions provided by the reviewers.

 Regarding comment number 7, the germ was only found on one slide HE, so PAS staining was done and showed aspects of actinomycosis with “spherical elements” most certainly sulfur granules. But as they were few in number and paraffin block almost insufficient we designated them only by “spherical cluster” (in Figure 3).

 And for comment number 8, we have checked the term "pre-perforative", which has been used in several scientific documents and articles. However, we remain open to any alternative terminology that may be proposed.

 Thank you, sincerely.
